# Investigation of Sonication Parameters for Large-Volume Focused Ultrasound-Mediated Blood–Brain Barrier Permeability Enhancement Using a Clinical-Prototype Hemispherical Phased Array

**DOI:** 10.3390/pharmaceutics16101289

**Published:** 2024-09-30

**Authors:** Dallan McMahon, Ryan M. Jones, Rohan Ramdoyal, Joey Ying Xuan Zhuang, Dallas Leavitt, Kullervo Hynynen

**Affiliations:** 1 Physical Sciences Platform, Sunnybrook Research Institute, Toronto, ON M4N 3M5, Canada; rmjones@sri.utoronto.ca (R.M.J.); khynynen@sri.utoronto.ca (K.H.); 2 Department of Medical Biophysics, University of Toronto, Toronto, ON M4N 3M5, Canada; 3 Institute of Biomedical Engineering, University of Toronto, Toronto, ON M5S 3G9, Canada

**Keywords:** focused ultrasound, microbubble, blood–brain barrier, infusion, image-guided therapy

## Abstract

**Background/Objectives:** Focused ultrasound (FUS) and microbubble (MB) exposure is a promising technique for targeted drug delivery to the brain; however, refinement of protocols suitable for large-volume treatments in a clinical setting remains underexplored. **Methods:** Here, the impacts of various sonication parameters on blood–brain barrier (BBB) permeability enhancement and tissue damage were explored in rabbits using a clinical-prototype hemispherical phased array developed in-house, with real-time 3D MB cavitation imaging for exposure calibration. Initial experiments revealed that continuous manual agitation of MBs during infusion resulted in greater gadolinium (Gd) extravasation compared to gravity drip infusion. Subsequent experiments used low-dose MB infusion with continuous agitation and a low burst repetition frequency (0.2 Hz) to mimic conditions amenable to long-duration clinical treatments. **Results:** Key sonication parameters—target level (proportional to peak negative pressure), number of bursts, and burst length—significantly affected BBB permeability enhancement, with all parameters displaying a positive relationship with relative Gd contrast enhancement (*p* < 0.01). Even at high levels of BBB permeability enhancement, tissue damage was minimal, with low occurrences of hypointensities on T2*-weighted MRI. When accounting for relative Gd contrast enhancement, burst length had a significant impact on red blood cell extravasation detected in histological sections, with 1 ms bursts producing significantly greater levels compared to 10 ms bursts (*p* = 0.03), potentially due to the higher pressure levels required to generate equal levels of BBB permeability enhancement. Additionally, albumin and IgG extravasation correlated strongly with relative Gd contrast enhancement across sonication parameters, suggesting that protein extravasation can be predicted from non-invasive imaging. **Conclusions:** These findings contribute to the development of safer and more effective clinical protocols for FUS + MB exposure, potentially improving the efficacy of the approach.

## 1. Introduction

Focused ultrasound (FUS) in combination with intravenously administered microbubbles (MBs) has emerged as a promising technique for non-invasively and transiently enhancing the permeability of the blood–brain barrier (BBB). This method has significant implications for targeted drug delivery to the brain, potentially improving the treatment of various neurological disorders. Despite the promising potential of this technique, optimization of sonication parameters and MB delivery strategies to maximize safety and efficacy over large treatment volumes in a clinical environment remains an understudied area.

A large body of literature from the past 20 years has provided evidence of efficacy in animal models of brain tumours [1] and Alzheimer’s disease [2,3,4], motivating early clinical trials to evaluate safety [5,6,7,8]. Since these studies, the next wave of trials has begun to evaluate efficacy, with promising results to date [9,10]. Additionally, an abundance of preclinical research has been directed towards optimizing sonication parameters, exposure feedback control strategies, and MB dosing to achieve safe and effective treatments (see review [11]). However, there is a sparsity of studies that have focused on tuning these parameters in the context of long-duration/large-volume clinical treatments. Chief amongst considerations to facilitate these types of treatments may be MB dose and delivery strategy.

Current Health Canada guidance restricts the total dose of Definity^TM^ for FUS + MB-mediated BBB permeability enhancement trials to 150 μL/kg [12], necessitating trade-offs between MB dose rate, treatment volume, and various sonication parameters. While parameters used in patient treatments to date have proven effective in generating BBB permeability enhancement across tissue volumes greater than 30 cm^3^ [10,13], room may exist for further optimization to achieve larger treatment volumes for drug delivery over a greater spatial extent. Additionally, MB delivery strategies for larger volume clinical treatments have often used gravity drip infusion with periodic mixing of saline–MB dilution during administration [10,12,13,14]. While this method of delivery has clear practical advantages, it may be prone to variance in MB size distribution and concentration over the course of administration due to MB segregation as a result of buoyancy [15]. This may in turn lead to variance in the bioeffects generated from sonication [16].

Additional considerations in designing sonication schemes for achieving large-volume sonications include the following: burst length, number of bursts, burst repetition frequency (BRF), and exposure level (i.e., peak negative pressure (PNP)). Minimizing burst length and/or number of bursts is conducive to faster treatment rates (i.e., volume per unit time). Lower BRFs (i.e., less than 1 Hz) allow for more complete reperfusion of MBs in vasculature within a given tissue volume [17] and have been shown to produce a greater magnitude of BBB permeability enhancement [18]. Use of higher exposure levels may permit equivalent magnitudes of drug delivery to be achieved over treatments with shorter total FUS ‘on-times’ (i.e., with fewer bursts or shorter bursts lengths, all else equal); however, this must be balanced with an increased risk of overt tissue damage at higher PNPs [13,19,20,21].

In this study, we aimed to evaluate the impact of various sonication parameters on BBB permeability enhancement and overt tissue damage, with an eye towards enabling large-volume treatments, using a clinical-prototype sparse transmit/receive hemispherical phased array FUS system. Based on the results of preliminary experiments comparing gravity drip to continuous manual agitation infusion, the latter was used in an effort to reduce the variability of FUS + MB-induced bioeffects resulting from long duration treatments. Additionally, real-time 3D MB cavitation imaging enabled exposures to be calibrated at each individual target based on the presence of spatially coherent MB activity. We provide insights into the effectiveness of this control strategy across a range of sonication parameters while using a low-dose MB infusion, an essential step prior to bringing the entirety of this treatment protocol and FUS device into the clinical testing phase. The results presented here could aid in the continued development of safe and effective FUS + MB protocols for clinical applications, potentially improving the delivery of therapeutic agents to the brain and advancing the treatment of neurological diseases.

## 2. Materials and Methods

### 2.1. Experimental Design

All animal procedures were approved by *Sunnybrook Research Institute’s* (SRI’s) *Animal Care Committee* and are in accordance with standards set by the *Canadian Council on Animal Care*. Experiments were performed on a total of 16 New Zealand White rabbits (male, 3–4 kg; Charles River, Saint-Constance, QC, Canada). FUS + MB exposure was performed up to three times in each animal, with a minimum of two weeks between sonications. MRI was performed immediately following sonication to assess the impact on BBB permeability enhancement and overt vascular damage. A preliminary set of experiments were performed on four rabbits (aka Cohort #1) to compare two MB infusion methods, gravity drip vs. continuous manual agitation, the latter being used for subsequent experiments. Cohort #2 (*n* = 12 rabbits) explored the impact of burst length, target level, and number of treatment (Tx) phase bursts (sonication parameters defined in Section 2.4) on gadolinium (Gd) contrast enhancement, indications of haemorrhage, and protein extravasation. A breakdown of the number of targets exposed with each combination of sonication parameters for Cohorts #1 and #2 can be found in Table 1. A subset of naive rabbits in Cohort #2 (*n* = 5) were sacrificed approximately 1.5 h following the start of their first FUS + MB exposure to assess red blood cell (RBC) and protein extravasation in tissue sections.

### 2.2. Animal Preparation

Animals were housed in SRI’s animal facility (Toronto, Canada) with access to food and water ad libitum. Induction of anaesthesia was facilitated by intramuscular administration of ketamine (50 mg/kg) and xylazine (5 mg/kg) to allow for tracheal intubation, after which anaesthesia was maintained with 1–3% isoflurane and oxygen or medical air (3–4 L/min). Medical air was used during MB infusion due to the impact of oxygen as a carrier gas on MB half-life in circulation [22,23]. Hair on the scalp was removed via electric clippers and depilatory cream, then washed with mild soap to prevent skin irritation. Angiocatheters (22G) were placed bilaterally in the lateral ear veins.

**Figure 1:**
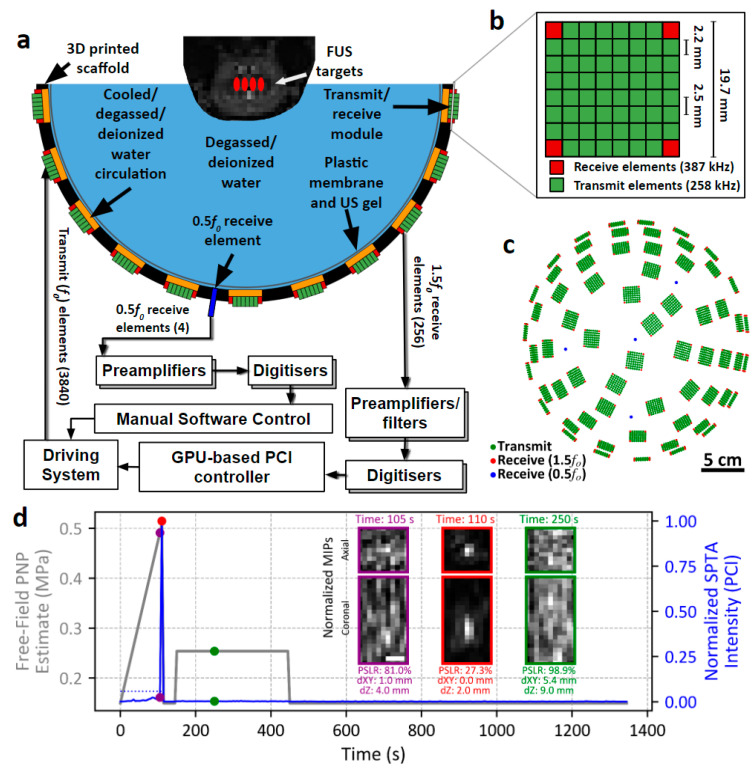
Clinical-prototype FUS array system and PCI-based cavitation feedback control example. (**a**) Experimental setup. Animals were positioned supine with scalps coupled directly to degassed/deionized water within the FUS array. Ultraharmonic receivers were used for PCI-based control and subharmonic receivers were used for ICD. (**b**) Transmit/receive module. Each module contains 64 transducer elements (8 × 8 grid, 2.5 mm inter-element spacing, 60 transmit and 4 receive elements). (**c**) Transmit and receive array layouts. (**d**) PCI-based cavitation feedback control example from in vivo data. PNP was iteratively increased until the detection of coherent MB activity via PCI. On the burst prior to detection (t = 105 s, magenta; PNP = 0.49 MPa) there was no evidence of coherent MB activity on PCI and the SPTA intensity remained below the maximum levels observed during baseline pressure ramps without MBs in circulation (blue dotted line). Spatially coherent MB activity observed in PCI MIPs (t = 110 s, red; calibration PNP = 0.51 MPa) was accompanied by a large spike in the SPTA intensity. The driving voltage was reduced to the minimum system output until the calibration phase was completed at all targets. In this example, a target level of 50% was set (Tx phase PNP = 0.25 MPa for 60 bursts). There was no evidence of MB activity on PCI throughout the Tx phase during which the SPTA intensity remained below the maximum levels observed during baseline pressure ramps, as seen at t = 250 s (green). White scale bar = 4 mm. FUS: focused ultrasound; ICD: inertial cavitation detection; MB: microbubble; PCI: passive cavitation imaging; PNP: peak negative pressure; SPTA: spatial peak temporal average; Tx: treatment.

For targeting scans and FUS + MB exposures, the animals were positioned supine on a platform with their heads fixed in place with custom-built ear/bite bars. Their heads were positioned such that their brains were in close proximity to the geometric centre of the FUS array with their scalps in contact with the degassed/deionized water to allow for acoustic coupling (Figure 1a). Animal temperature was maintained throughout imaging and the FUS procedure using a heated water circulation blanket. Heart rate, O_2_ saturation, and end tidal CO_2_ were continuously monitored (3880 MRI Patient Monitor, IRadimed Corp., Winter Springs, FL, USA).

### 2.3. Clinical-Prototype FUS Brain System

An in-house designed and manufactured transmit/receive (3840 transmit elements/256 receive elements) sparse hemispherical phased array system was used to deliver ultrasound, with transducer modules similar to those previously described [24,25]. Briefly, the FUS array consists of 64 modules secured in place by a 3D-printed (Accura Clearvue/ProJet 7000; 3D Systems, Rock Hill, SC, USA) hemispherical scaffold with a 25.1 cm diameter. Each module contains 64 rectangular cuboid lead zirconate titanate (PZT-4) elements arranged in an 8 × 8 grid with a 2.5 mm inter-element spacing (Figure 1b). Prior to array fabrication, multi-layered ray acoustic simulations [26] were performed to assess the impact of transducer module placement on the resulting array’s transmit performance, with a view towards suppressing grating/side lobe formation. Once module placement was determined on the basis of transmit simulations (Figure 1c), receive simulations were carried out to ensure transcranial imaging performance was acceptable with this module configuration [27]. Ray-tracing aberration corrections [28] adapted to account for acoustic refraction effects were employed during both transmit and receive simulations. The transducer modules were housed in 3D-printed casings (Accura Clearvue/ProJet 7000; 3D Systems, Rock Hill, SC, USA) designed to allow temperature controlled, deionized water to continuously circulate and degas within 4 mm deep reservoirs that interface with the active surface of each module; a thin mylar membrane allows ultrasound to propagate through this reservoir, into the degassed, deionized water that fills the FUS array.

**Figure 2:**
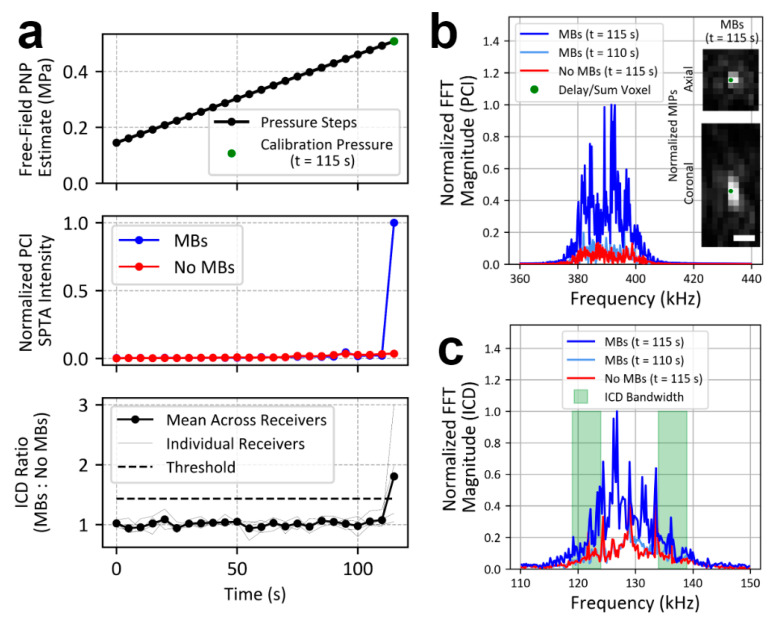
Calibration phase cavitation feedback control scheme example. (**a**) An in vivo example of the cavitation feedback control scheme used during the calibration phase of FUS + MB exposures. PNP was increased each burst until satisfying PCI detection thresholds and/or exceeding the threshold for ICD ratio (**top panel**). In this example, PCI detection thresholds were satisfied on the 24th burst of the calibration phase (time = 115 s, green marker), corresponding to a divergence in PCI SPTA intensity versus the baseline pressure ramp (i.e., no MBs in circulation; **middle panel**). The ICD threshold was exceeded during the same burst (**bottom panel)**. (**b**) Frequency spectrum of filtered (8th order digital Butterworth filter, 380–400 kHz bandpass) RF data delay-and-summed to the voxel of maximum PCI SPTA intensity (green marker) for the calibration pressure burst (t = 115 s, blue line; PNP = 0.51 MPa), as well as that of the same voxel and sonicating PNP during a baseline pressure ramp without MBs in circulation (t = 115 s, red line; PNP = 0.51 MPa). The filtered delay-and-summed frequency spectrum for the burst prior to the calibration pressure with MBs in circulation is also shown (t = 110 s, light blue line; PNP = 0.49 MPa). (**c**) The mean unfiltered frequency spectrum across 4 subharmonic receivers used for ICD is shown for the calibration pressure burst (t = 115 s; blue line; PNP = 0.51 MPa), the same sonicating PNP during a baseline pressure ramp without MBs in circulation (t = 115 s; red line; PNP = 0.51 MPa), and the burst prior to the calibration pressure with MBs in circulation (t = 110 s; light blue line; PNP = 0.49). Light green rectangles indicate the bandwidth used for ICD calculations. White scale bar = 4 mm.

The 4 corner elements (2.2 × 2.2 × 4.3 mm) of each module were tuned to the first ultraharmonic (387 kHz) of the driving frequency and used to receive acoustic emissions to control exposures via passive cavitation imaging (PCI). Fixed gain pre-amplifiers (40 dB) were incorporated into the receive chain and RF signals were digitized using sixteen 16-channel data acquisition cards (ATS9416; AlazarTech, QC, Canada). The remaining 60 elements (2.2 × 2.2 × 6.0 mm) within each module were driven at their lateral mode [29,30] resonance frequency of 258 kHz. Each individual transmit element was driven by a miniature linear amplifier and waveform generator developed in-house, which allows for programmable phase and amplitude. Receive element locations were confirmed using acoustic triangulation [31]. Four additional rectangular receivers (PZT-5, 4.7 × 4.7 × 11.0 mm) with an independent receive chain were distributed within the remaining space on the scaffold between transmit/receive modules; these receivers were tuned to the subharmonic (129 kHz) of the driving frequency and designed for detection of wideband signals (Figure 2c), indicative of inertial cavitation [32].

### 2.4. FUS Exposure Protocol

For animals in Cohort #1, 12 or 16 targets were positioned using MR images acquired with the FUS array and animal in the bore of the scanner (56 targets across 4 FUS + MB exposures in 4 rabbits). For Cohort #2, 16 targets were placed for each sonication (288 targets across 18 FUS + MB exposures in 13 rabbits). In both cohorts, targeted brain regions consisted largely of bilateral striatum and midbrain. Targets were placed initially as a 3 × 4 or 4 × 4 grid oriented in an axial plane, with a 3.5 mm spacing; individual targets were shifted to avoid specific structures (e.g., ventricles, subarachnoid cisterns, cerebellum, etc.). Ultrasound was delivered in 1, 5, or 10 ms bursts, electronically steered to target coordinates in sequence using a raster pattern starting at the posterior left-most target (Cohort #1: target-wise BRF = 1.0 Hz, Cohort #2: target-wise BRF = 0.2 Hz).

Vials of Definity^TM^ MBs (Lantheus Medical Imaging, North Billerica, MA, USA) were allowed to reach room temperature prior to activation (Vial-Mix; Lantheus Medical Imaging, North Billerica, MA, USA), after which the agent was extracted from an inverted vial using a 18 gauge needle while venting with a second 18 gauge needle [33,34]. For each cohort, intravenous infusion (1.6 μL/kg/min; 0.5 mL/min infusion rate of saline–MB solution) of Definity^TM^ was initiated 15 min prior to the start of sonication in order to allow adequate time to approach a steady-state concentration in circulation [18]. Infusion continued until the end of sonication. The mean total duration of MB infusion was 18.6 min ± 1.0 min for Cohort #1 and 37.7 min ± 0.5 min for Cohort #2 (variation arises from the variable duration of calibration phases between animals). For gravity drip infusion, MBs were diluted in a 100 mL saline bag. Drip rate was measured continuously using an infusion rate monitor (DripAssist; Shift Labs, Seattle, WA, USA) and adjusted to maintain 0.5 mL/min. The saline–MB solution was gently mixed at approximately 5 min intervals. “Continuous manual agitation infusion” consisted of repeated inversion/~180° rotation of a 50 mL syringe containing the saline–MB mixture (i.e., along the short axis of the syringe) in an effort to achieve a more consistent MB size distribution and concentration over time. 

A 3D MB cavitation imaging-based feedback approach [35] combined with independent inertial cavitation detection (ICD) were used to calibrate the in situ pressure level at each target. Sonications consisted of two stages; an initial “calibration phase”, in which the pressure threshold for detection of spatially coherent MB cavitation activity or inertial cavitation was determined (also known as calibration pressure), followed by a “Tx phase”. To determine the calibration pressure, during the calibration phase the PNP was increased (starting pressure ≈ 0.15 MPa, step size ≈ 15 kPa; free-field estimates) after each burst at a given target (Figure 2a, upper panel) until the detection of either: (1) spatially coherent MB emissions via PCI (Figure 2b) or (2) an increase in wideband emissions relative to baseline pressure ramps performed in the absence of MBs via ICD (Figure 2a, lower panel). Once spatially coherent MB emissions or inertial cavitation were detected, the peak-to-peak transmit voltage (V_pp_) was reduced to the minimum system output (0.55 V_pp_, ≈0.15 MPa) at that target for the remainder of the calibration phase. The Tx phase was initiated immediately following completion of the calibration phase at all targets. During the Tx phase, each target was sonicated at a fixed pressure proportional to the calibration pressure; treatment PNP = calibration PNP × target level (%) (Cohort #1: 50–90% target level, Cohort #2: 50% or 75% target level) for 0, 60, 120, or 240 Tx phase bursts. The sonication parameters investigated in Cohort #2 were designed to cover the range of values that have typically been used in the field [18,35,36,37,38].

As previously described [35,39], 3D PCI was performed on bandpass filtered receiver signals (zero-phase digital 8th order Butterworth, 380–400 kHz bandpass) using a delay, sum, and integrate beamforming algorithm over a 11 mm × 11 mm × 21 mm field of view (FOV) centred on the targeted coordinate, using a 1.0 mm × 1.0 mm × 1.0 mm voxel size (integration time = 1, 5, or 10 ms, corresponding to the entire burst length; capture length = 13.1 ms for all burst lengths, sampling rate = 10 MS/s). Thresholds for defining coherent MB activity were: peak side-lobe ratio (PSLR) ≤ 70%; and distance between the location maximum spatial-peak temporal-average (SPTA) PCI intensity and target coordinates ≤ 2.0 mm in the lateral plane; and ≤4.0 mm in the axial direction. These thresholds were determined using PCI maximum intensity projections (MIPs) collected during prior in vivo FUS array characterization in rabbits with MBs in circulation. PCI MIPs were manually classified as containing coherent MB activity or not. Precision-recall values were generated for different combinations of threshold parameters, with the combination that maximized recall while producing precision above 99.5% being chosen for exposure control moving forward. The false positive rate in the current study on baseline sonications (i.e., without MBs in circulation) using this set of thresholds was 0.12% (17/13,824) of bursts and 3.13% (9/288) of targets.

The thresholds for defining inertial cavitation, monitored with 4 independent receivers, were set in relation to baseline pressure ramps collected prior to MB administration (i.e., no MBs in circulation). For each receiver, the ratio of a spectral FFT integration (bandwidth: 119–139 kHz excluding a 10 kHz notch centred around subharmonic; Figure 2c) in the calibration phase to that in the baseline pressure ramps were calculated at each pressure step, then averaged across receivers (Figure 2a, lower panel). Channel-mean ratios exceeding 5 standard deviations (SDs) above the mean of the first 10 steps in the calibration phase resulted in a given target being flagged for the system operator to manually end the calibration phase for that target.

In Cohort #2, targets were excluded from subsequent analyses if: (1) the effective target level exceeded intended target level by more than 5% based on retrospective observation of coherent MB activity in the calibration phase that failed to meet PCI detection thresholds (*n* = 9), (2) coherent MB activity was retrospectively observed in the Tx phase (*n* = 32), (3) PCI detection thresholds were reached, but retrospective manual classification of PCI MIPs indicated a false positive detection (*n* = 4), and/or (4) quantification of relative Gd contrast enhancement was complicated by ventricles or subarachnoid cisterns (*n* = 13). In total, 51 of 288 targets were excluded from subsequent analyses.

### 2.5. MRI and Quantification

All sonications were performed under MRI guidance with the animal and FUS array within the bore of a 3.0T MRI scanner (Biograph mMR, Siemens Healthcare, Erlangen, Germany). A 3D T2-weighted (T2w) sequence was used to acquire images used for coregistration of MRI and FUS array coordinates based on the locations of fiducial markers fixed at known locations within the FUS array, as well as for subsequent target placement within the brain. Following FUS + MB exposures, animals were transferred to the MRI bed and images were acquired (11 cm loop coil) with a T2*-weighted (T2*w) sequence to evaluate RBC extravasation and a T1-weighted (T1w) sequence to assess extravasation of a Gd-based contrast agent. To assess BBB permeability enhancement, a dose of Gadovist was administered (0.1 mL/kg, IV bolus; Bayer Inc., Toronto, ON, Canada) both at the start of the calibration phase and prior to post-FUS + MB imaging. In Cohort #1, the second dose of Gadovist for gravity drip infusion and continuous manual agitation infusion was administered 12–13 min and 31–34 min, respectively, following the start of sonication. For Cohort #2, the second dose of Gadovist was administered 33.7 ± 1.9 min following the start of sonication. The parameters for each MRI sequence are detailed in Table 2.

To assess extravasation of Gd, T1w signal intensity within circular regions of interest (ROIs; 6 voxel/2.3 mm diameter) centred on each target was normalized to that of non-sonicated regions in a slice-wise manner (Figure A2). ROIs were placed by two authors (D.M. and R.M.J.), blinded to treatment conditions, and relative Gd contrast enhancement values were averaged between them. For each target, the maximum relative Gd contrast enhancement value across all imaging planes was used for subsequent analyses. Post-sonication images acquired with T2*w MRI were qualitatively assessed for the presence of hypointensities, indicative of microhemorrhage.

### 2.6. Histological Processing and Analysis

A subset of rabbits in Cohort #2 (*n* = 5) were sacrificed approximately 1.5 h following the start of their first FUS + MB exposure to assess RBC and protein extravasation in tissue sections. Animals were transcardially perfused with saline, followed by 10% neutral buffered formalin. Brains were extracted and post-fixed in 10% neutral buffered formalin, then paraffin embedded. Groups of axial sections (5 μm thick) were collected at 500 μm intervals; one series of sections was hematoxylin-eosin (H&E)-stained and another was used for immunofluorescence.

Three H&E-stained sections per animal, estimated to be centred at the plane displaying maximum relative Gd contrast enhancement in T1w images, were imaged with a 10× objective lens using brightfield microscopy (Axio Imager 2, Zeiss, Göttingen, Germany). Summed area of RBC extravasation in each section was determined using a Weka classifier [40] trained to detect RBCs. The model used the following features: Gaussian blur, Sobel filter, hessian, and difference of Gaussians. The membrane patch size was set to 19 and membrane thickness to 1. The minimum and maximum sigma values were set equal to 1 and 8, respectively. A random forest classifier (numFeatures = 2; numTrees = 60) was used. The maximum tree depth was determined automatically. On validation data, the model exhibited an accuracy of 99.5% versus manual segmentation. For each target, the maximum RBC segmented area across the image’s sections was used for subsequent analyses.

Adjacent sections were immunostained for rabbit serum albumin and IgG. Sections were deparaffinized, washed (0.1 M phosphate buffer, 0.1% triton-X, 0.1% tween-20), and antigen retrieval was performed in citrate buffer (10 mM, pH 6) under pressure and heat (114 °C for 20 min; 103 °C for 20 min). Sections were washed three times and blocked (0.1 M phosphate buffer, 0.1% triton-X, 0.1% tween-20, 5% normal goat serum, 1% bovine serum albumin) for 30 min at room temperature. Incubation was performed overnight at 4 °C using sheep anti-rabbit albumin antibody conjugated to FITC (1:100; A120-104F, Thermofisher, Waltham, MA, USA) and goat anti-rabbit IgG antibody conjugated to Alexa568 (1:100; A-11036, Thermofisher, Waltham, MA, USA). Washed sections were coverslipped with aqueous mounting media (Fluoroshield with DAPI, Sigma-Aldrich, St. Louis, MO, USA) and stored in the dark at 4 °C until imaging. The immunostained sections were imaged with an epifluorescence microscope (Axio Imager 2, Zeiss, Göttingen, Germany) using DAPI, DsRed-IgG, and green fluorescent (GFP)-albumin filters, and a 10× objective lens.

Fluorescent intensity in circular ROIs centred on each target were measured in the DsRed and GFP channels and normalized to background signal intensity in non-sonicated areas of brain tissue; measurements were performed on each target and section in ImageJ. For each target, the maximum relative signal intensity value across the imaged sections was used for subsequent analyses.

### 2.7. Statistics

For analyses of relative Gd contrast enhancement in Cohort #1, two-way Student’s *t*-test was used. For Cohort #2 analyses, analysis of variance (ANOVA) with post-hoc Tukey’s honestly significant difference (HSD) test was used to assess the impact of sonication parameters on relative Gd contrast enhancement. Correlations for relative Gd contrast enhancement vs. RBC extravasation area and relative Gd contrast enhancement vs. relative immunofluorescent signal intensities were assessed using linear regression. The effects of sonication parameters on RBC extravasation area and relative immunofluorescent signal intensities were analyzed using ANOVA with and without relative Gd contrast enhancement as a covariate. Tukey’s HSD test was used post-hoc to assess group differences. A *p*-value of 0.05 was used as the threshold for statistical significance. Variance is expressed as standard deviation of the mean.

## 3. Results

### 3.1. Preliminary Evidence for Impact of MB Infusion Method on BBB Permeability Enhancement

**Figure 3:**
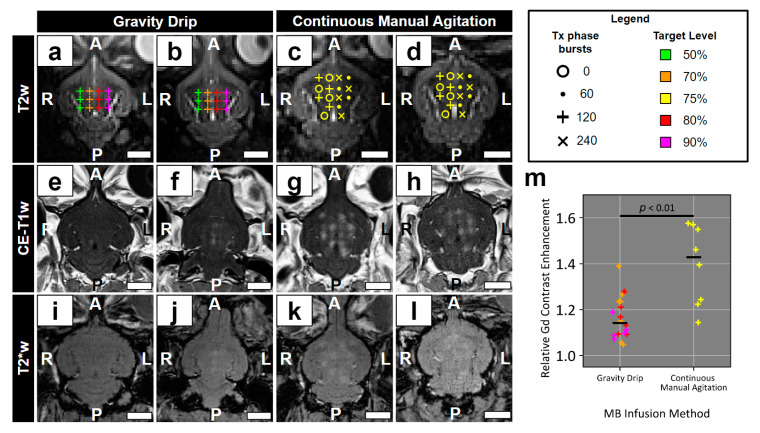
Preliminary comparison of MB infusion methods. In a subset of rabbits (n = 4; aka Cohort #1), the impact of MB infusion method on BBB permeability enhancement was evaluated. (**a**–**d**) Target layout and sonication parameters are displayed in relation to T2w targeting scans for gravity drip (n = 24 targets) and continuous manual agitation (n = 32 targets) infusion methods. (**e**–**h**) T1w MRI highlights differences in relative Gd contrast enhancement between infusion methods and various sonication parameters. (**i**–**l**) No evidence of hypointensities in T2*w MRI were observed. (**m**) Relative Gd contrast enhancement is plotted for gravity drip and continuous manual agitation infusion; for each infusion method, only targets for which target level ≥ 70% and Tx phase bursts = 120, were considered (n = 8 targets for continuous manual infusion; n = 18 targets for gravity drip infusion). A significant difference was detected between infusion methods (*p* < 0.01). White scale bars = 1 cm. A: anterior; L: left; P: posterior; R: right.

In a small subset of rabbits (*n* = 4; aka Cohort #1), the impact of two MB infusion methods on relative Gd contrast enhancement was assessed as a pilot study (Figure 3). When comparing targets with similar sonication parameters (i.e., target level ≥ 70%, Tx phase bursts = 120, burst length = 5 ms, BRF = 1 Hz), continuous manual agitation (*n* = 8 targets) produced significantly greater levels of relative Gd contrast enhancement compared to gravity drip (*n* = 18 targets) infusion (continuous manual agitation: 1.43 ± 0.16, gravity drip: 1.14 ± 0.09, *p* < 0.01). Although the sample size is low, the large effect size motivated the use of continuous manual agitation infusion for experiments moving forward. All subsequent results are described in relation to Cohort #2 experiments.

### 3.2. Dual-Strategy Acoustic Emissions-Based Exposure Calibration In Vivo

The combined PCI- and ICD-based approach for exposure level calibration was successful in detecting cavitation activity in the brain during all sonications carried out with MBs in circulation. Spatially coherent MB activity was detected via 3D PCI during the calibration phase of 87% of targets (206/237). An example of 3D MB imaging-based calibration is shown in Figure 1d. Once MB concentration in circulation was estimated to have reached a steady-state, the calibration phase was initiated and the applied PNP level was increased burst-by-burst until coherent MB activity was detected near this specific target (Figure 1d). Detection of coherent MB activity typically coincided with an increase in the SPTA PCI image intensity both relative to the previous burst and to baseline sonications at equivalent exposure levels but without MBs in circulation, as shown in this example (Figure 1d). Once the calibration phase was completed for all targets, the PNP level at this target was fixed to 50% of its calibration pressure (i.e., 50% target level) for the Tx phase of the sonication. No coherent MB activity was detected during the Tx phase at this target (Figure 1d), which is consistent with the findings from sonications carried out in rabbits using a different transmit/receive FUS device [41] at similar target levels while monitoring at the subharmonic frequency during the Tx phase [35,39].

Wideband emissions were detected via ICD during the calibration phase of 49% of targets (116/237). Spatially coherent MB activity was detected simultaneously via PCI during the same burst for which wideband emissions were detected in the majority of these targets (85/116 targets). An example of concurrent cavitation detection is provided in Figure 2, where spatially coherent MB activity on PCI (Figure 2b) and wideband emissions on ICD (Figure 2c) occurred during the same burst. For 13% of targets (31/237), exposure level calibration was carried out using wideband emission detection via ICD without any indications of spatially coherent MB activity observed with PCI. A summary of the calibration pressure levels and PCI image quality metrics from in vivo calibration phase sonications is provided in Table 3, as reported for previous work using a different prototype transmit/receive FUS device [35,39]. Significant differences in calibration PNP for 1 ms vs. 10 ms burst lengths (*p* < 0.01) and 5 ms vs. 10 ms burst lengths (*p* < 0.01) were observed (Table 3).

### 3.3. Impact of Sonication Parameters on Relative Gd Contrast Enhancement

**Figure 4:**
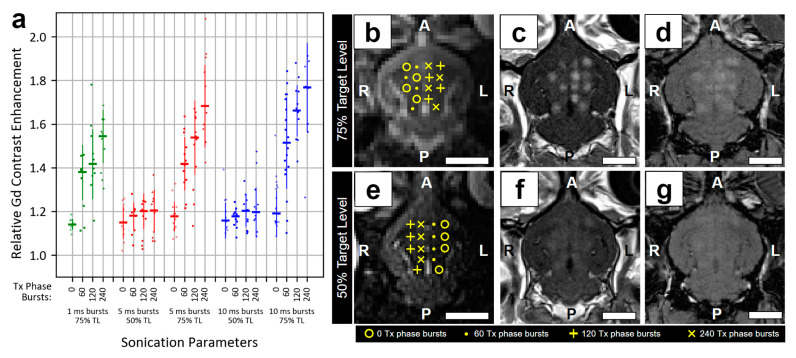
Relative Gd contrast enhancement across sonication parameters. T1w MRI was performed approximately 10 min following the end of sonication. (**a**) Target-wise (n = 237) relative Gd contrast enhancement is plotted for each set of sonication parameters investigated. Burst length (*p* < 0.01), target level (*p* < 0.01), and number of Tx phase bursts (*p* < 0.01) had significant effects on relative Gd contrast enhancement. Targets sonicated with 75% target level exhibited significantly greater levels of relative Gd contrast enhancement vs. 50% target level (*p* < 0.01). At 75% target level, burst length had a significant impact on relative Gd contrast enhancement (*p* < 0.01), with a significant difference between 1 ms vs. 10 ms burst lengths (*p* = 0.048). Number of Tx phase bursts also had a significant effect on relative Gd contrast enhancement at 75% target level (*p* < 0.01). TL = target level. Representative examples of the targeting scheme (**b**,**e**), Gd contrast enhancement in T1w MRI (**c**,**f**), and T2*w MRI (**d**,**g**) are shown for sonications performed with 5 ms bursts and either 75% (**b**–**d**) or 50% (**e**–**g**) target levels. Number of Tx phase bursts range from 0 to 240 within each animal. White scale bars = 1 cm. A: anterior; L: left; P: posterior; R: right.

The magnitude of BBB permeability enhancement was quantified by relative Gd contrast enhancement in T1w MRI collected approximately 10 min following the end of sonication (Figure 4c,f). Burst length (*p* < 0.01), target level (*p* < 0.01), and number of Tx phase bursts (*p* < 0.01) all had a significant effect on relative Gd contrast enhancement (Figure 4a). Targets sonicated with 75% target level exhibited significantly greater levels of relative Gd contrast enhancement vs. 50% target level (50% target level: 1.18 ± 0.08, 75% target level: 1.43 ± 0.24, *p* < 0.01). At 75% target level, burst length had a significant effect on relative Gd contrast enhancement (*p* < 0.01); post-hoc tests indicated a significant difference between 1 ms and 10 ms burst lengths (1 ms: 1.36 ± 0.18, 10 ms: 1.48 ± 0.25, *p* = 0.048), but not between 1 ms and 5 ms (5 ms: 1.43 ± 0.24, *p* = 0.35) or 5 ms and 10 ms burst lengths (*p* = 0.55).

At a 75% target level, the number of Tx phase bursts had a significant effect on relative Gd contrast enhancement (*p* < 0.01); post-hoc tests indicated significant differences between all paired comparisons (*p* < 0.01) except 60 vs. 120 Txt phase bursts (*p* = 0.05) and 120 vs. 240 Tx phase bursts (*p* = 0.06) (0 bursts: 1.19 ± 0.09; 60 bursts: 1.44 ± 0.18; 120 bursts: 1.53 ± 0.18; 240 bursts: 1.64 ± 0.20). No interaction effect was observed between burst length and number of bursts in the Tx phase (*p* = 0.1). Burst length (*p* = 0.20) and number of Tx phase bursts (*p* = 0.36) did not significantly influence relative Gd contrast enhancement at 50% target level. Results indicate that under these experimental conditions (i.e., MB infusion method/dose, control strategy, etc.), a target level > than 50% and >0 Tx phase bursts are required to generate a clinically relevant level of relative Gd contrast enhancement using burst lengths of 1–10 ms.

### 3.4. T2*w MRI Rarely Displayed Signal Hypointensities

Following sonication, T2*w MRI was completed for each animal to assess haemorrhage. Observation of T2*w signal hypointensities was rare. Of the 237 targets included in all analyses, 2 displayed signal hypointensities indicative of haemorrhage. The sonication parameters for these two targets were 10 ms burst length/75% target level/240 Tx phase bursts (1/8) and 1 ms burst length/75% target level/120 Tx phase bursts (1/10). A total of 104 targets included in all analyses were performed with a target level of 75% and 60 or more Tx phase bursts.

Of the 51 targets excluded from analyses, 7 displayed hypointensities on T2*w MRI. Of these targets, six exhibited coherent MB activity in the Tx phase and one was located in a subarachnoid cistern, complicating evaluation. All seven targets were sonicated with 240 Tx phase bursts and 75% target level. Overall, a low probability of damage was observed under sonication conditions that produced high levels of relative Gd contrast enhancement, provided coherent MB activity was not observed in the Tx phase.

### 3.5. RBC Extravasation Correlated with Relative Gd Contrast Enhancement

**Figure 5:**
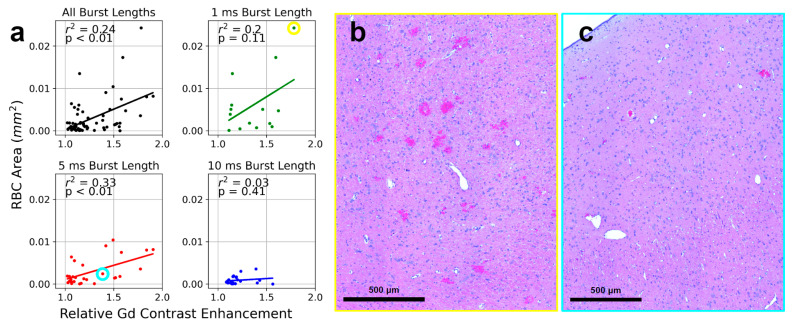
RBC extravasation area across sonication parameters. In a subset of rabbits, the area of RBC extravasation was quantified in H&E-stained tissue sections (66 targets, 5 rabbits). The animals were perfused 1.5 h following the start of FUS + MB exposure. (**a**) A significant correlation between relative Gd contrast enhancement and RBC area was observed across all sonication parameters (r^2^ = 0.24, *p* < 0.01). For targets sonicated with 5 ms bursts a significant correlation was also observed (r^2^ = 0.33, *p* < 0.01). At 75% target level, when relative Gd contrast enhancement was considered as a covariate, burst length (*p* < 0.01) had significant effects on RBC extravasation. Post-hoc Tukey’s HSD test revealed a significant difference between 1 ms and 10 ms burst lengths (*p* = 0.03). (**b**) The target displaying the largest area of RBC extravasation observed (yellow border) was sonicated with 1 ms bursts, 75% target level, and 120 Tx phase bursts. This target displayed hypointense signal intensity on T2*w imaging (Figure A3). The data point corresponding to this target is circled (yellow) in panel (**a**). (**c**) A histological image representative of 5 or 10 ms burst lengths and 75% target level is displayed (cyan border). Low levels of RBC extravasation are observed across the sonicated volume. The data point corresponding to this target is circled (cyan) in panel (**a**).

In a subset of naive animals (*n* = 5) sacrificed approximately 1.5 h following the start of sonication, overt vascular damage was assessed in H&E-stained sections (66 targets). The area of RBC extravasation was found to be significantly correlated to relative Gd contrast enhancement across all sonication conditions (r^2^ = 0.24, *p* < 0.01, Figure 5a). For targets sonicated with 1 ms, 5 ms, and 10 ms burst lengths, r^2^ values of 0.20 (*p* = 0.11), 0.33 (*p* < 0.01), and 0.03 (*p* = 0.41), respectively, were observed.

Across all the sonication parameters, burst length had a significant effect on the area of RBC extravasation (1 ms: 0.006 mm^2^ ± 0.007 mm^2^, 5 ms: 0.003 mm^2^ ± 0.003 mm^2^, 10 ms: 0.0008 mm^2^ ± 0.0009 mm^2^, *p* = 0.01). Post-hoc analysis revealed significant differences for comparisons of 1 ms vs. 5 ms (*p* = 0.04) and 1 ms vs. 10 ms (*p* < 0.01).

For targets sonicated with a 75% target level, when relative Gd contrast enhancement was considered as a covariate, the number of Tx phase bursts (*p* < 0.01; Figure A4) and burst length (*p* < 0.01; Figure 5a) had significant effects on RBC extravasation. Post-hoc analysis revealed a significant difference between 1 ms and 10 ms burst lengths (*p* = 0.03); this indicates that at equal levels of relative Gd contrast enhancement, 1 ms bursts lead to significantly greater levels of RBC extravasation compared to 10 ms bursts.

In the subset of targets included in histological analysis, one displayed evidence of signal hypointensity on T2*w MRI (Figure A3). This target was sonicated using 1 ms burst length, 75% target level, and 120 bursts in the Tx phase, and was observed to have the greatest area of RBC extravasation across histological sections (Figure 5b). Overall, at targets that did not display evidence of signal hypointensity on T2*w MRI, the area of RBC extravasation was generally observed to be low, but had some relationship to relative Gd contrast enhancement and sonication parameters.

### 3.6. Extravasation of Blood-Borne Proteins Correlated with Relative Gd Contrast Enhancement

**Figure 6:**
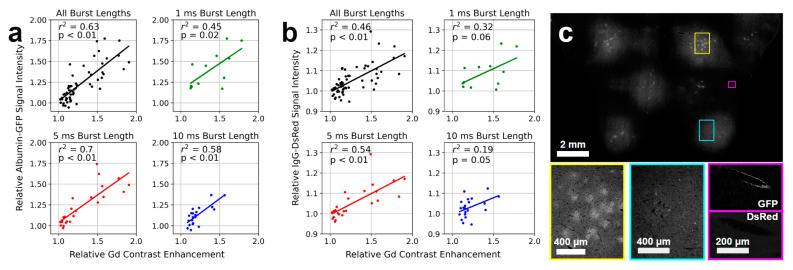
Albumin and IgG immunofluorescence following sonication. In a subset of animals, immunofluorescent staining for albumin and IgG was performed on tissue sections from rabbits perfused for 1.5 h following the end of FUS + MB exposure (5 rabbits, 66 targets). A strong linear correlation was observed between relative Gd contrast enhancement and relative immunofluorescent signal intensity for both (**a**) albumin–GFP (r^2^ = 0.63, *p* < 0.01) and (**b**) IgG-DsRed (r^2^ = 0.46, *p* < 0.01). Across all sonication parameters, the target level had a significant effect on relative immunofluorescent signal intensity for both (**a**) albumin–GFP (*p* < 0.01) and (**b**) IgG-DsRed (*p* < 0.01). (**a**,**b**) When relative Gd contrast enhancement was considered as a covariate, no significant effect of target level or number of Tx phase bursts on relative immunofluorescent signal intensity of either protein were observed. (**c**) A representative example of albumin–GFP immunofluorescence for 8 posterior targets sonicated with 1 ms burst lengths and 75% target level is displayed. Areas of relatively homogeneous signal intensity across the target volume (cyan border) is contrasted with a more heterogeneous signal intensity (yellow border). Evidence of perivascular transport of albumin–GFP is shown in an area distant from any targeted volume (magenta border); IgG-DsRed signal intensity is not higher than background levels in this ROI.

Immunofluorescent staining for albumin and IgG in brain tissue sections, a measure of BBB permeability enhancement, was performed on the same subset of animals used for RBC extravasation quantification (*n* = 5). Across all sonication parameters (66 targets), a strong linear correlation was observed between relative Gd contrast enhancement and relative immunofluorescent signal intensity for both albumin–GFP (r^2^ = 0.63, *p* < 0.01, Figure 6a) and IgG-DsRed (r^2^ = 0.46, *p* < 0.01, Figure 6b). For targets sonicated with 1 ms, 5 ms, and 10 ms burst lengths, r^2^ values of 0.45 (*p* = 0.02), 0.7 (*p* < 0.01), and 0.58 (*p* < 0.01), respectively, were observed for the correlation between relative Gd contrast enhancement and relative albumin–GFP signal intensity. Likewise, for targets sonicated with 1 ms, 5 ms, and 10 ms burst lengths, r^2^ values of 0.32 (*p* = 0.06), 0.54 (*p* < 0.01), and 0.19 (*p* = 0.05), respectively, were observed for the correlation between relative Gd contrast enhancement and relative IgG-DsRed signal intensity.

Across all sonication parameters, target level had a significant effect on relative immunofluorescent signal intensity for both albumin–GFP (*p* < 0.01) and IgG-DsRed (*p* < 0.01). Post-hoc analysis revealed significant differences between 50% and 75% target levels for both proteins of interest (albumin–GFP: 50% target level = 1.07 ± 0.09, 75% target level = 1.33 ± 0.20, *p* < 0.01; IgG-DsRed: 50% target level = 1.01 ± 0.04, 75% target level = 1.08 ± 0.07, *p* < 0.01). The number of Tx phase bursts had a significant impact on relative immunofluorescent signal intensity of albumin–GFP (*p* < 0.01); however, no significant differences were observed between any pairwise comparisons. When relative Gd contrast enhancement was considered as a covariate, no significant effects of target level or number of Tx phase bursts (Figure A5) on relative immunofluorescent signal intensity of either protein were observed.

Similarly, for targets sonicated with a 75% target level, no significant relationship was observed between relative albumin–GFP intensity and burst length or the number of Tx phase bursts when relative Gd contrast enhancement was considered as a covariate. The same was true for relative IgG-DsRed intensity. This indicates that relative Gd contrast enhancement explains much of the variance in relative immunofluorescent signal intensities for albumin and IgG.

Qualitative differences were observed in the homogeneity of albumin–GFP and IgG-DsRed signal intensity between targets. Some targets displayed immunofluorescent signal distribution indicative of relatively uniform protein extravasation (Figure 6c cyan inset), while others displayed more punctate regions extravasation (Figure 6c yellow inset); however, no clear relationship between immunofluorescent signal homogeneity and sonication parameters were observed. Additionally, consistent with previous reports [42,43,44,45], albumin–GFP immunofluorescence was observed surrounding large vessels outside of targeted tissue (Figure 6c magenta inset, upper panel), indicative of perivascular transport of extravasated proteins 1.5 h after the end of sonication. The same was not observed for IgG-DsRed (Figure 6c magenta inset, lower panel).

## 4. Discussion

FUS + MB-mediated BBB permeability enhancement has emerged as a promising technique for facilitating targeted drug delivery to the brain. Despite its potential, studies seeking to optimize sonication parameters for safe, efficacious treatments over large treatment volumes and in a clinically relevant manner are lacking. Here, the impact of various sonication parameters and two infusion methods were explored using a clinical-prototype hemispherical phased array FUS system, real-time 3D MB cavitation imaging for exposure calibration, and low-dose MB infusion in rabbits. The results indicate that a high degree of BBB permeability enhancement can be achieved without producing MRI indications of overt tissue damage using sonication parameters that allow for the treatment of large volumes. This work also demonstrates the effect of several sonication parameters on the magnitude of BBB permeability enhancement generated, which may serve to better control drug delivery and risk of unwanted tissue damage in a clinical setting.

Preliminary experiments on a small subset of rabbits demonstrated a large impact of the MB infusion method on subsequent BBB permeability enhancement, with continuous manual agitation generating a significantly greater level of relative Gd contrast enhancement than gravity drip infusion. This motivated the use of continuous manual agitation infusion in all experiments moving forward. Largely, preclinical and clinical research in the field has utilized MB administration techniques that are not well suited to long-duration/large-volume sonications. These include bolus administration [5,6,8,46,47], short-duration and relatively high-concentration infusions [18,35,48], and low-concentration gravity drip infusions [10,12,13,14]. To achieve large-volume treatments with a low risk of MB-related adverse events, the dose rate and total dose of MB administered must be carefully considered. Currently, the maximum allowable dose of DefinityTM MBs approved by Health Canada for FUS + MB-mediated BBB permeability enhancement trials is 150 μL/kg [12]. With this limit, the administration rate used in the current study (1.6 μL/kg/min) would allow for just over 90 min of MB delivery time, providing a sufficient treatment window to target large tissue volumes (e.g., 5 ms burst length, ~20 calibration phase bursts, 60 Tx phase bursts, 15 min infusion time prior to start of first sonication, 0.2 Hz BRF, 30% duty cycle → 3300+ targets treated → 70+ cm3 treatment volume with 2.2 mm × 2.2 mm × 4.4 mm grid spacing).

Additional considerations for MB delivery strategy include the stability of in vivo concentration over time and MB size distribution administered. While bolus administration may be conducive to achieving a consistent MB size distribution at the time of delivery, the use of repeated boluses for large-volume treatments will likely suffer from large in vivo concentration fluctuations or may necessitate long intervals between administrations for MB concentration to return to baseline levels [17,49]. The former is likely to contribute to variance in the bioeffects generated from FUS + MB exposures [16,50], whereas the latter will lead to longer procedure times. Conversely, gravity drip infusion, a method used in several clinical trials to date [10,12,13,14], is a practical solution for delivering MBs over long treatment durations; however, the size distribution of MBs delivered over the course of treatment may vary without continual agitation [51], as gas-filled microspheres segregate at a rate approximately proportional to the square of their diameter [15]. Even with periodic mixing of the MB–saline solution, MBs may have the opportunity to segregate in the vertical tubing that extends to the intravenous catheter. This also has the potential to impact the bioeffects generated over the course of a treatment [16], although proposed control strategies that modulate FUS exposure output to achieve a prescribed cavitation dose may be better suited to account for the changes in MB size distribution or concentration over time [12,13,18,52]. When PNP is increased, the strength of the scattered acoustic signals will also generally increase due to larger amplitude MB oscillations, thus potentially leading to greater bioeffects. In the current study, repeated inversions of the syringe/MB–saline solution during infusion (aka continuous manual agitation) was performed with the goal of achieving a more uniform in vivo concentration and size distribution over time, compared to gravity drip infusion. Based on the preliminary results reported here, future work should be directed towards a more thorough characterization of infusion methods used in MB-mediated FUS therapies and the development of an infusion pump that allows for continuous agitation of a MB solution in an MRI environment.

**Figure A1:**
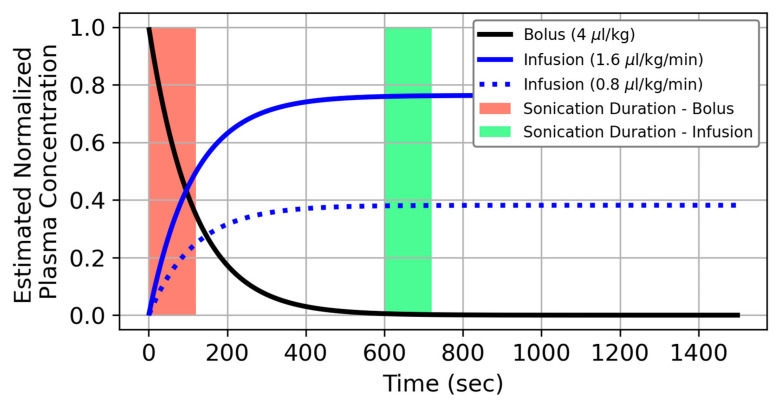
Comparison of estimated plasma concentration of Definity^TM^ MBs over time. One compartment model of MB concentration in circulation over time for bolus (4 μL/kg) and infusion (0.8 μL/kg/min and 1.6 μL/kg/min) administration. Half-life of Definity^TM^ was assumed to be 79 s [57]. For bolus administration, t = 0 represents the peak concentration in circulation. For infusion administration, t = 0 represents the start of delivery. Sonication durations are set to 120 s.

**Figure A2:**
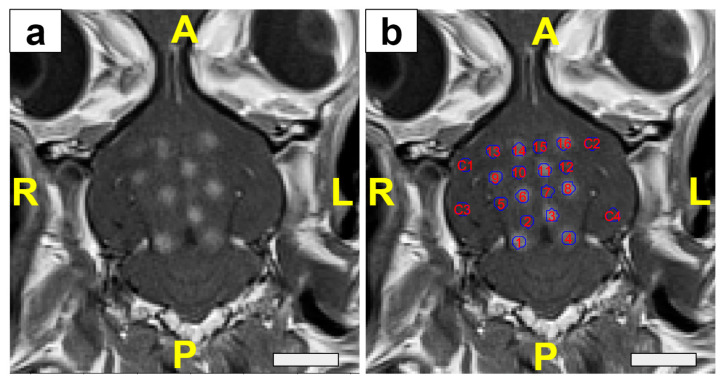
Relative Gd contrast enhancement analysis. (**a**) An example of contrast enhanced T1w MRI collected following FUS + MB exposure. (**b**) ROIs selected for quantification of T1w signal intensity at targets (#1–16), as well as regions used as non-sonicated control tissue (C1–C4). Mean signal intensity within each targeted ROI was divided by mean signal intensity across the non-sonicated control ROIs to obtain relative Gd contrast enhancement values. White scale bars = 1 cm. A: anterior; L: left; P: posterior; R: right.

**Figure A3:**
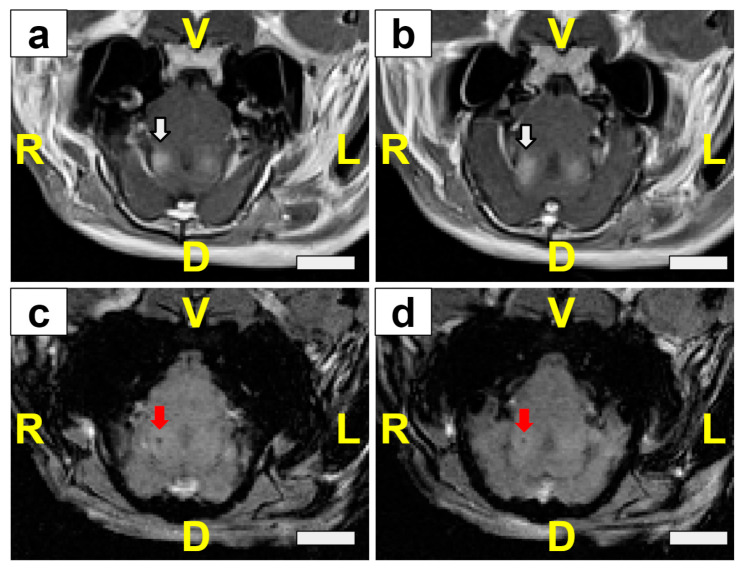
T2*w hypointensity induced by FUS + MB exposure. (**a**,**b**) Sequential coronal slices from contrast enhanced T1w MRI collected following FUS + MB exposure. White arrows highlight a single target of increased BBB permeability. (**c**,**d**) Red arrows highlight a small area of hypointensity in T2*w MRI corresponding to the same target highlighted above. This target displayed the largest area of RBC extravasation in H&E sections across all targets processed for histology. White scale bars = 1 cm. D: dorsal; L: left; R: right; V: ventral.

**Figure A4:**
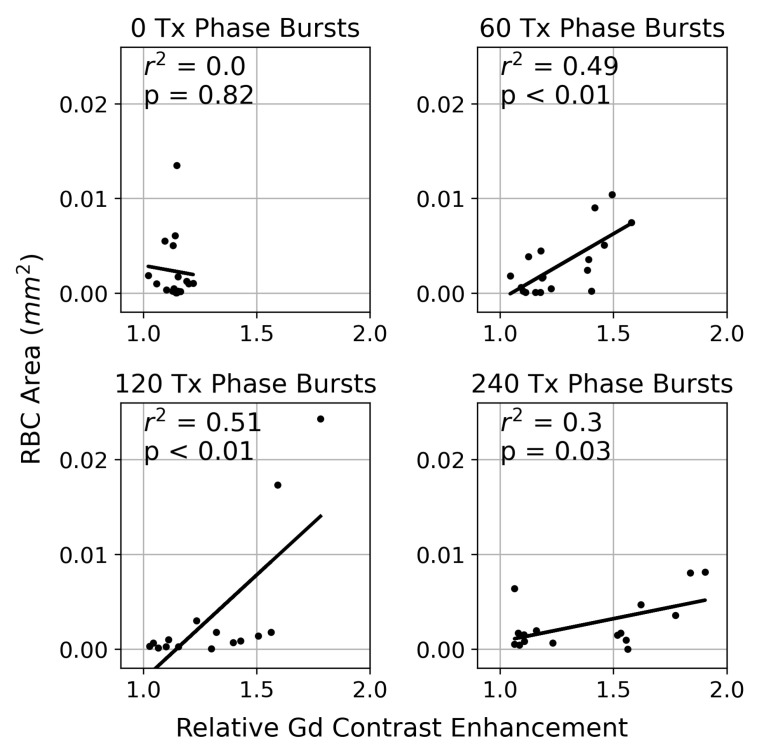
Correlations between RBC extravasation area and relative Gd contrast enhancement for different numbers of Tx phase bursts. In a subset of rabbits, area of RBC extravasation was quantified in H&E stained tissue sections (66 targets, 5 rabbits). Animals were perfused 1.5 h following the end of FUS + MB exposure. A significant correlation between relative Gd contrast enhancement and RBC area was observed across all sonication parameters (r^2^ = 0.24, *p* < 0.01). For targets sonicated with 60, 120, and 240 Tx phase bursts, significant correlations were also observed (60 Tx phase bursts: r^2^ = 0.49, *p* < 0.01; 120 Tx phase bursts: r^2^ = 0.51, *p* < 0.01; 240 Tx phase bursts: r^2^ = 0.3, *p* = 0.03). For targets sonicated with 75% target level, when relative Gd contrast enhancement was considered as a covariate, number of Tx phase bursts (*p* < 0.01) and burst length (*p* < 0.01; Figure 5a) had significant effects on RBC extravasation. Post-hoc analysis revealed no significant differences between any paired comparison of Tx phase burst numbers.

**Figure A5:**
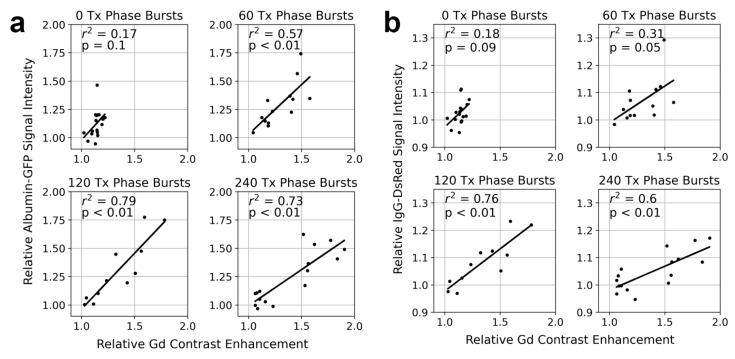
Correlations between protein immunofluorescence and relative Gd contrast enhancement for different numbers of Tx phase bursts. In a subset of animals, immunofluorescent staining for albumin and IgG was performed on tissue sections from rabbits perfused 1.5 h following the end of FUS + MB exposure (5 rabbits, 66 targets). A strong linear correlation was observed between relative Gd contrast enhancement and relative immunofluorescent signal intensity for both (**a**) albumin-GFP (r^2^ = 0.63, *p* < 0.01) and (**b**) IgG-DsRed (r^2^ = 0.46, *p* < 0.01). When targets sonicated with an equal number of Tx phase bursts were considered, significant correlations were observed for 60, 120, and 240 Tx phase bursts for both albumin-GFP (60 Tx phase bursts: r^2^ = 0.57, *p* < 0.01; 120 Tx phase bursts: r^2^ = 0.79, *p* < 0.01; 240 Tx phase bursts: r^2^ = 0.73, *p* < 0.01) and IgG-DsRed (60 Tx phase bursts: r^2^ = 0.31, *p* = 0.05; 120 Tx phase bursts: r^2^ = 0.76, *p* < 0.01; 240 Tx phase bursts: r^2^ = 0.60, *p* < 0.01). (**a**,**b**) When relative Gd contrast enhancement was considered as a covariate, no significant effect of the number of Tx phase bursts on relative immunofluorescent signal intensity of either protein were observed.

As has been previously reported, the MB dose has a large influence on the bioeffects generated from FUS + MB exposures [16,50,53]. While the desire for larger volume/longer duration clinical treatments motivates the use of progressively lower doses of MBs to stay below current regulatory safety limits, it is important to consider the potential impact this may have on treatment outcomes. Initial clinical trials for transcranial FUS + MB-mediated BBB permeability enhancement employed bolus administration of DefinityTM at a dose of 4 μL/kg [5,8]; trials employing gravity drip infusion have often administered 0.8 μL/kg/min or lower [10,12,13,14]. When considering the short half-life of DefinityTM, the estimated concentration of MBs in circulation during sonications differ substantially under these regimes, with 4 μL/kg bolus administration sustaining a higher plasma concentration for almost the entirety of the sonication compared to 0.8 μL/kg/min MB infusion (Figure A1). For the current study, 1.6 μL/kg/min MB infusion was employed to more closely mimic the MB concentration achieved in the initial trials that employed bolus administration.

Following Cohort #1 experiments, the impact of key sonication parameters was explored in the context of low-dose MB infusion using continuous manual agitation. Of the sonication parameters explored in this work, target level (i.e., target level × calibration pressure = Tx phase pressure) had the largest impact on BBB permeability enhancement. Relative Gd contrast enhancement exhibited a more than 2-fold increase when comparing 50% to 75% target levels across sonication parameters. Burst length and number of Tx phase bursts also had a significant impact on relative Gd contrast enhancement, with longer burst lengths and a greater number of Tx phase bursts generally leading to a greater degree of BBB permeability enhancement. Despite achieving a high level of Gd contrast enhancement at a 75% target level, indications of damage on T2*w MRI were rarely observed (2/104 targets). Histological examination of a subset of rabbits 1.5 h following the end of sonication revealed small areas of RBC extravasation that exhibited a low but significant correlation to the degree of relative Gd contrast enhancement observed on T1w MRI. The variables explaining the remaining variance are unclear, however, the low correlation may suggest that higher levels of BBB permeability may be achieved without necessarily increasing the risk of vascular damage.

The low levels of RBC extravasation observed at this acute time point would be expected to be cleared in the days following sonication, as has previously been observed [13,35]. Analysis of targets sonicated with a 75% target level indicated that, even accounting for relative Gd contrast enhancement and number of Tx phase bursts, burst length had a significant impact on RBC extravasation, with 1 ms bursts being associated with a greater level of extravasation compared to 10 ms bursts. This result is in line with previous observations of greater vascular damage associated with short pulses at equal levels of relative Gd contrast enhancement compared to longer tone bursts [44]. All else being equal, shorter burst lengths likely require higher PNPs to generate an equivalent level of BBB permeability enhancement, leading to a greater risk of inducing vascular damage. In the current study, shorter burst lengths required a significantly higher PNP for the detection of coherent MB activity or inertial cavitation (Table 3), but produced lower levels of BBB permeability enhancement (Figure 4a).

Extravasation of albumin and IgG under different sonication parameters followed a similar trend as that of relative Gd contrast enhancement. A higher target level and greater number of Tx phase bursts led to a greater degree of protein extravasation. Much of the variance in protein extravasation across sonication conditions could be explained by the variance in relative Gd contrast enhancement; longer bursts or a greater number of Tx phase bursts did not appear to drive larger molecules into neuropil more than what would be predicted by relative Gd contrast enhancement. This suggests that albumin and IgG extravasation following FUS + MB exposures, and by extension drugs with similar properties, can be reasonably estimated from non-invasive imaging across the range of sonication parameters explored in this work. Similar conclusions have been drawn from studies employing more uniform sonication parameters [54,55].

Additionally, qualitative evaluation of albumin immunofluorescence revealed evidence of perivascular transport 1.5 h following the end of sonication. Strong signal intensity was observed surrounding large vessels distant from the targets of sonication (Figure 6c magenta inset, upper panel). This was not observed for IgG (Figure 6c magenta inset, lower panel). Evidence for glymphatic transport of extravasated molecules following FUS + MB exposure has been reported in several studies to date [42,43,44,45]; however, the authors believe this to be the first report indicating variation in the transport kinetics of different molecules following sonication (i.e., albumin vs. IgG). It is possible that differences in mass (albumin, 67 kDa; IgG, 150 kDa) may contribute to different rates of transport [56].

In determining sonication parameters suitable for clinical translation, one must consider risks and benefits associated with the treatment. In general, a greater magnitude of BBB permeability enhancement is associated with a greater risk of generating overt tissue damage and inducing a more pronounced acute inflammatory response [50,57,58]. Under the conditions used for Cohort #2 of the current study (i.e., acoustic control strategy, MB dose, MB infusion method, FUS system, etc.), FUS + MB exposures employing 75% target level, 5–10 ms burst lengths, and 60–120 Tx phase bursts, were found to consistently generate substantial increases in BBB permeability without MRI indication of overt tissue damage.

For clinical translation of large-volume treatments, it is also important to consider target spacing. The axial in-plane inter-point spacing employed in this work (3.5 mm, or 0.6 λ with λ the acoustic wavelength) was intentionally large to avoid overlapping targets and facilitate target-wise MRI and histological analyses. In clinical treatment scenarios, the inter-point spacing is reduced to achieve more spatially homogeneous patterns of elevated BBB permeability. Results of a previous preclinical study using a higher-frequency (612 kHz) prototype hemispherical FUS system suggested an inter-point spacing of approximately 0.4 λ produced a better compromise between BBB opening volume and enhancement uniformity [35], which corresponds to 2.3 mm at the driving frequency employed in the current work (258 kHz). Initial clinical trials of FUS-mediated BBB permeabilization using a commercial low-frequency (220–230 kHz) transcranial FUS brain system have employed inter-point spacings consistent with this finding (2.5–3.0 mm, or 0.35–0.45 λ) [5,8,12,13]. It is worth noting that there may be risks of unwanted tissue damage associated with sonications carried out with overlapping targets caused by small inter-point spacings (e.g., 0.15 λ in [59]).

### Limitations

One limitation of the current study is that following the initial calibration phase, Tx phase sonications were performed at a fixed pressure level, without online feedback control. Retrospective analysis of the acoustic emissions data revealed that 32/288 targets displayed evidence of coherent MB activity during the Tx phase, with the majority of these events being associated with a higher target level (29 at 75% target level, 3 at 50% target level). Previous work from our group using a similar PCI-based control strategy with a different transmit/receive FUS phased array did not observe coherent subharmonic MB activity in the Tx phase at the 50% target level [35,39]. These prior studies employed shorter duration, higher concentration MB infusions and sonications with shorter Tx phases (2 min vs. up to 20 min in the present work), which may explain these differences. Observations from the current study may suggest a change in target volume MB concentration and/or size distribution over time, which may contribute to the detection of coherent MB activity at pressures below the initial calibration pressure. This may be the result of local changes in vascular perfusion [60] and/or systemic changes. Given that a large proportion of targets exhibiting hypointensities on T2*w MRI displayed evidence of coherent MB activity in the Tx phase, there is a clear need for feedback control throughout the entire sonication duration, which will be incorporated in future studies.

Another limitation of this work is the small number of animals included in the histological and immunofluorescence analyses. A total of five rabbits were sacrificed 1.5 h after the end of sonication, one from each combination of target level and burst length. The conclusions drawn from these analyses are in line with the previous literature; however, inter-animal variation cannot be accounted for in these data. Thus, some degree of caution is warranted in generalizing results from these analyses. Similarly, the comparison of gravity drip to continuous manual agitation infusion was conducted on a total of four rabbits with differences in sonication parameters between groups. To account for the differences in target level and number of Tx phase bursts, only targets that were sonicated using target levels ≥ 70% and 120 tx phase bursts were included in this analysis. This means that, despite the average target level of the targets included in analysis for the gravity drip infusion group being higher than that of the continuous manual agitation group, the mean relative Gd contrast enhancement was greater for the latter group. While the effect size was large enough to motivate the use of the continuous manual agitation infusion method in subsequent experiments, future work should more thoroughly explore this result in a greater number of animals and with identical sonication parameters. Additionally, while the Gd contrast administration timing differed between the two groups, the earlier administration time point for the gravity drip group should favour a greater level of relative Gd contrast enhancement compared to the continuous manual agitation group [61].

Finally, the present study was performed through the intact rabbit skull, without employing element-specific aberration corrections. While the relatively thin skull bones of the rabbit combined with the low transmit frequency used here limit the expected degradation of transcranial focusing, in a clinical setting there is a greater need for aberration corrections due to the increased thickness of human cranial bones relative to the acoustic wavelength(s) of interest. Experiments in a large animal model with ex vivo human skullcaps and non-invasive aberration corrections on both transmit and receive (i.e., simulation-based using computed tomography (CT)-derived skull morphology [27,62] or microbubble emission-based [63,64]) are warranted prior to clinical testing.

## 5. Conclusions

FUS + MB-mediated BBB permeability enhancement shows substantial promise for facilitating targeted drug delivery to the brain, though optimization of the sonication parameters is crucial for maximizing efficacy and safety across large treatment volumes in a clinical setting. This study underscores the significant impact of various sonication parameters—specifically target level, burst length, and number of Tx phase bursts—on BBB permeability enhancement and associated risks. Notably, high levels of relative Gd contrast enhancement were achieved using a clinical-prototype hemispherical phased array FUS system and a MB administration strategy conducive to large-volume treatments, with minimal indications of tissue damage. The insights gleaned from this work will guide future clinical trials with the goal of further improving the efficacy and safety of FUS + MB-mediated drug delivery in patients.

## Figures and Tables

**Table 1:** Sonication Parameters.

Cohort	MB Infusion Method	Sonication Parameters				Number of Targets Sonicated	Number of Animals
		Burst Length (ms)	Target Level (%)	Number of Tx Phase Bursts	Target-Wise BRF (Hz)		
1	Continuous manual agitation (1.6 μL/kg/min)	**5**	75	0	1.0	8	2
		5	75	60		8	
		5	75	120		8	
		5	75	240		8	
	Gravity drip (1.6 μL/kg/min)	5	50	120		6	2
		5	70	120		6	
		5	80	120		6	
		5	90	120		6	
2	Continuous manual agitation (1.6 μL/kg/min)	1	75	0	0.2	12	3
		1	75	60		12	
		1	75	120		12	
		1	75	240		12	
		5	50	0		12	3
		5	50	60		12	
		5	50	120		12	
		5	50	240		12	
		5	75	0		16	4
		5	75	60		16	
		5	75	120		16	
		5	75	240		16	
		10	50	0		12	3
		10	50	60		12	
		10	50	120		12	
		10	50	240		12	
		10	75	0		20	5
		10	75	60		20	
		10	75	120		20	
		10	75	240		20	

BRF: burst repetition frequency; MB: microbubble; Tx: treatment.

**Table 2:** MRI parameters.

	T2w (Targeting)	T1w (Post-FUS + MBs)	T2*w (Post-FUS + MBs)
**Sequence type**	3D SPACE	TSE	3D GRE
**Echo time (ms)**	239	8.6	15
**Repetition time (ms)**	5000	616	27
**Number of averages**	2	3	2
**FOV (mm)**	163 × 163	100 × 100	100 × 100
**Matrix size**	192 × 192	256 × 256	256 × 256
**Slice Thickness (mm)**	1.5	1.5	1.5
**Flip angle (°)**	120	150	13

FOV: field of view; FUS: focused ultrasound; GRE: gradient echo; MB: microbubble; SPACE: Sampling Perfection with Application optimized Contrast using different flip angle Evolution; T1w: T1 weighted; T2w: T2 weighted; T2*w: T2* weighted; TSE: turbo spin echo.

**Table 3:** Calibration pressure and PCI image quality metrics. The calibration PNP values are free-field estimates. Significant differences in calibration PNP for 1 ms vs. 10 ms burst lengths (*p* < 0.01) and 5 ms vs. 10 ms burst lengths (*p* < 0.01) were observed. Steering distance denotes the total distance from the target location to the FUS array’s geometric focus, and the corresponding steering factor was estimated based on numerical ray acoustic simulations. Positional error is defined as the total distance between the location of SPTA PCI intensity and the intended target. The main lobe beamwidths (short/long axis sizes) are calculated based on 3D ellipsoidal fits of the −3 dB PCI intensity isosurfaces.

Metric	Number of Bursts Included	Mean ± SD	Range [Min, Max]
Calibration PNP (MPa)			
All burst lengths	237	0.53 ± 0.09	[0.38, 0.94]
1 ms burst length	38	0.58 ± 0.08	[0.45, 0.82]
5 ms burst length	95	0.55 ± 0.10	[0.38, 0.94]
10 ms burst length	104	0.50 ± 0.07	[0.38, 0.74]
Intra-Grid Calibration PNP Range (MPa)	237	0.23 ± 0.09	[0.10, 0.45]
Calibration Time (s)	237	117 ± 26	[70, 235]
Steering Distance (mm)	237	28 ± 6	[17, 39]
Steering Factor (%)	237	91 ± 2	[87, 94]
PSLR (%)	206	49 ± 13	[18, 70]
Positional Error (mm)	206	2.0 ± 1.0	[0, 4.2]
−3 dB Main Lobe Short Axis (mm)	197	2.2 ± 0.3	[1.6, 3.4]
−3 dB Main Lobe Long Axis (mm)	197	5.4 ± 1.0	[3.8, 9.0]

PNP: peak negative pressure; PSLR: peak side-lobe ratio; SD: standard deviation.

## Data Availability

The raw data supporting the conclusions of this article may be made available by the authors on request.

## Abbreviations

ANOVA: analysis of variance; BBB: blood–brain barrier; BRF: burst repetition frequency; FFT: fast Fourier transform; FOV: field of view; FUS: focused ultrasound; Gd: gadolinium; GFP: green fluorescent protein; GRE: gradient echo; H&E: hematoxylin-eosin; HSD: honestly significant difference; ICD: inertial cavitation detection; IgG: immunoglobulin G; IV: intravenous; MB: microbubble; MIP: maximum intensity projection; MRI: magnetic resonance imaging; PCI: passive cavitation imaging; PNP: peak negative pressure; PSLR: peak side-lobe ratio; PZT-4: lead zirconate titanate; RBC: red blood cell; ROI: region of interest; SD: standard deviation; SPACE: Sampling Perfection with Application optimized Contrast using different flip angle Evolution; SPTA: spatial-peak temporal-average; SRI: Sunnybrook Research Institute; T1w: T1-weighted; T2w: T2-weighted; T2*w: T2*-weighted; TSE: turbo spin echo; Tx: treatment; V_pp_: peak-to-peak transmit voltage.
